# *Curcuma longa* L. Water Extract Improves Dexamethasone-Induced Sarcopenia by Modulating the Muscle-Related Gene and Oxidative Stress in Mice

**DOI:** 10.3390/antiox10071000

**Published:** 2021-06-23

**Authors:** Shintae Kim, Kyungmi Kim, Jeongjin Park, Woojin Jun

**Affiliations:** 1 Division of Food and Nutrition, Chonnam National University, Gwangju 61187, Korea; rotoman1@naver.com; 2 Department of Biofood Analysis, Korea Bio Polytechnic, Ganggyung 32946, Korea; kkm64@kopo.ac.kr; 3 Research Institute for Human Ecology, Chonnam National University, Gwangju 61187, Korea

**Keywords:** sarcopenia, *Curcuma longa* L., ladder climbing exercise, muscle function, antioxidants

## Abstract

Dexamethasone (DEX) promotes proteolysis, which causes muscle atrophy. Muscle atrophy is connected to sarcopenia. We evaluated the effect of *Curcuma longa* L. water extract (CLW) on DEX-induced muscle atrophy. ICR mice were divided into three groups (eight mice per group) to investigate the capability of CLW in inhibiting muscle atrophy. The control group (Ex-CON) was administered distilled water (DW) by gavage and subjected to exercise; the muscle atrophy group (Ex-DEX) was administered DW by gavage, an injection of DEX (1 mg/kg body weight/day) intraperitoneally (IP), and subjected to exercise; and the treatment group (Ex-CLW) was administered CLW (1 g/kg body weight/day) by gavage, DEX IP injection, and subjected to exercise. Following the injection of DEX, the expression levels of myostatin, MuRF-1, and Atrogin-1 were increased. However, these expression levels were decreased in the Ex-CLW group, thereby leading to the conclusion that CLW inhibits muscle atrophy. ROS (that was overproduced by DEX) decreased antioxidant enzyme activity and increased malondialdehyde (MDA) levels, which led to muscle atrophy. When CLW was ingested, the antioxidant enzyme activities increased while the MDA levels decreased. These findings suggest that CLW could serve as a natural product for the prevention of muscle atrophy by modulating muscle atrophy-related genes and increasing antioxidant potential.

## 1. Introduction

Muscle atrophy refers to a decrease in muscle mass that is caused by aging, a lack of exercise, or diseases, thereby resulting in a faster rate of protein degradation than the rate of protein synthesis [1]. Protein degradation is caused by autophagy and the ubiquitin proteasome system [1]. Autophagy is a process that naturally breaks down unnecessary or nonfunctional cell components, resulting in the formation of autophagosomes. The resulting autophagosomes and lysosomes are fused and decomposed [2]. In the ubiquitin proteasome system, a proteasome recognizes and decomposes misfolded or unnecessary proteins attached to ubiquitin. Muscle-specific ligases include muscle ring finger 1 (MuRF-1) and muscle atrophy F box (Atrogin-1). Their overexpression is known to promote muscle atrophy [3]. Myostatin regulates the expression of MuRF-1 and Atrogin-1 [4]. Inhibiting the expression of myostatin causes muscles to become hypertrophic. The expression of MuRF-1 and Atrogin-1 is increased to breakdown muscle proteins and prevent muscle growth. Muscle atrophy also results from increased oxidative stress [5].

Several methods have been used to induce muscle atrophy in animal models. Methods that cause muscle atrophy include unloading [6], immobilization [7], starvation [8], denervation [9], and injection of glucocorticoids [10]. From these, we opted to use the glucocorticoid injection method. Glucocorticoids are widely used as an anti-inflammatory agent. However, their long-term use produces several side effects. One of the side effects of glucocorticoids is muscle atrophy [11]. Dexamethasone (DEX) is a type of glucocorticoid that promotes proteolysis, which causes muscle atrophy [10]. DEX also increases oxidative stress, which leads to muscle atrophy [12]. When DEX is used, the ubiquitin proteasome system is activated during proteolysis [13]. Myostatin is also activated by DEX, thereby increasing MuRF-1 and Atrogin-1 expression, resulting in muscle atrophy [14].

Exercise includes resistance and aerobic exercises. Aerobic exercise improves cardiorespiratory endurance, such as walking, jogging, swimming, and running [15], while resistance exercise involves increasing resistance against the muscle work, which helps improve muscle strength and mass [16]. In several studies, strength training was demonstrated to suppress muscle atrophy [17,18,19].

*Curcuma longa* L. belongs to the ginger family and is native to South Asia. *C. longa* is known as the plant from which curry powder is made. [20]. The rhizome, the portion of the plan used medicinally, yields a yellow powder [21]. *C. longa* contains curcumin and has many health benefits including a detoxifying effect on the liver [21], pain relief for arthritis and muscle pain [22], anticancer properties [23], and anti-inflammatory properties [24]. Curcumin has been used as an oral supplement in the management of various medical conditions [25,26,27]. Other studies have demonstrated that *C. longa* induces mitochondrial biosynthesis in muscle and promotes smooth muscle differentiation [28,29]. Mitochondria are important organelles that produce energy in muscles. Aging reduces mitochondrial density and muscle mass [30]. However, maintaining or increasing the number of mitochondria can prevent muscle loss [31]. Additionally, myosatellite cells recover when muscles are damaged. After creating myoblasts from myosatellie cells, they are differentiated into myotubes [32]. Thus, the ingestion of *C. longa*, which promotes mitochondrial biogenesis by increased cAMP and muscle differentiation by increased cell proliferation, may suppress muscle atrophy.

In the present study, the inhibitory effect of water extract of *C. longa* (CLW) on muscle atrophy was evaluated in the DEX-induced mouse model. Its underlying mechanism relevant to muscle atrophy was investigated via an examination of the expression of muscle metabolic genes and proteins.

## 2. Materials and Methods

### 2.1. Sample and Chemicals

The hot water extract of *C. longa* that was grown in Jindo Province, Jeollanamdo was provided by SDC (Damyang, Korea). The *C. longa* powder was dissolved into 20 folds water at 250 °C for 3 h. The extract was filtered, vacuum evaporated, and then lyophilized. It was stored at −20 °C for further experiments. Hydroperoxide, 1,1,3,3-tertramethoxypropane, thiobarituric acid, trichloroacetic acid, xanthine, xanthine oxidase, superoxide dismutase (SOD), catalase (CAT), glutathione peroxidase (GPx), and β-nicotinamid adenine dinucleotide phosphate reduced form (NADPH) were obtained from Sigma-Aldrich Co. (St. Louis, MO, USA). All other chemicals used were of analytical grade.

### 2.2. Animal Experiments

Eight-week-old ICR mice were acclimatized for 1 week prior to the start of the experiment. The mice were housed in a temperature and humidity of 23 °C and 50%, respectively, and were subjected to a 12 h day and night cycle. All mice were fed a 5L79 diet and distilled water. All experimental animals were raised and tested after receiving approval from the Chonnam National University Institutional Animal Care and Use Committee (CNU IACUC-YB-R-2019-91).

The mice were allocated into the following three groups based on weight and grip strength (8 mice per group): (1) Ex-CON, mice administered distilled water (DW) by gavage and exercise; (2) Ex-DEX, mice administered DW by gavage, intraperitoneally (IP) injected with DEX (1 mg/kg body weight/day), and exercise; (3) Ex-CLW, mice administered CLW (1 g/kg body weight/day) by gavage, IP injected with DEX, and exercise. All the groups participated in the ladder climbing exercise. DW and CLW was administered by gavage daily from 1 week before the DEX injection. DEX was IP injected for 14 days. The ladder climbing exercise was performed once every 2 days. At the end of the experiment, the mice were anesthetized by isoflurane. Blood samples were collected transcardially via the apex of the left ventricle. The tissue samples were collected and stored at −80 °C for further analysis.

### 2.3. Ladder Climbing Exercise

The dimensions of the ladder were 100 × 7 × 5 cm (height × width × depth) with an angle of 70°. First, the exercise was performed without additional weight on the mouse’s tail. Thereafter, 50% of the mouse’s body weight was attached onto the mouse’s tail and the ladder was adjusted to enable climbing. The weight was gradually increased until 100% of the weight was reached. The ladder climbing exercise took a total of 8 round trips. Between the rounds, the exercising mice were allowed to rest for 1 min. The ladder climbing exercise was conducted once every 2 days.

### 2.4. Grip Strength

A grip strength meter (Bio-GS3, Bioseb, Vitrolles, France) was used to measure the force immediately before the mice fell off the grid by placing the mice on the grid and pulling their tail. Grip strength was measured once a week and repeated three times to obtain an average value.

### 2.5. Evaluation of Muscle Histopathology

The muscles of the mice were fixed in formalin. After a paraffin block was generated and the muscle tissue was cut, hematoxylin and eosin (H&E) staining was performed on a slide glass. The dyed muscle tissue was observed by ×200 magnification with a microscope (Leica, Wetzler, Germany). The cross-sectional area (CSA) of 300 muscle fibers was measured using ImageJ (NIH, Bethesda, MD, USA) software.

### 2.6. Real-Time Polymerase Chain Reaction

After homogenizing the muscle tissue with liquid nitrogen, the total RNA was extracted using easy-BLUE^TM^ (INtRON Biotechnology, Seongnam, Korea) according to the easy-BLUE^TM^ manual. The quantitative RNA was synthesized with cDNA, and a real-time polymerase chain reaction (RT-PCR) was performed with the cDNA. The primer sequences were as follows; myostatin: 5′-CCTCCACTCCGGGAACTGA-3′, 5′- AAGAGCCATCACTGCTGTCATC-3′; MuRF-1: 5′-TCTGCCTGGAGATGTTTACCAA-3′, 5′-TCCGGCAGAGGTTGTGTTG-3′; Atrogin-1: 5′-AAGGGCAGCTGGATTGGAA-3′, 5′-GGTGACCCCATACTGCTCTCTT-3′; β-actin: 5′-ACGGCCAGGTCATCACTATTG-3′, 5′-CAAGAAGGAAGGCTGGAAAAGA-3′. The mRNA expression level was calculated using the ∆∆CT method.

### 2.7. Western Blotting

Muscle tissue was extracted with protein using the RIPA lysis buffer (Invitrogen, Carlsbad, CA, USA). The extracted protein was quantified with the Bradford assay. The amount of protein extracted from each group was equalized to 50 μg and then electrophoresed using SDS-PAGE. After the electrophoretic SDS-PAGE gel was transferred to nitrocellulose membranes, it was incubated with the primary antibody overnight at 4 °C. The primary antibodies used were MuRF-1 (1:1000, Invitrogen, Carlsbad, CA, USA), atrogin-1 (1:1000, Invitrogen, Carlsbad, CA, USA), and β-actin (1:1000, Invitrogen, Carlsbad, CA, USA). After the primary antibody reaction, a secondary antibody (horseradish peroxidase-labeled anti-rabbit, 1:10,000, Invitrogen, Carlsbad, CA, USA) was add for 1 h. For protein signal intensity, a Clarity Western ECL substrate kit (Bio-Rad, Hercules, CA, USA) was employed. ChemiDoc XRS+ (Bio-Rad, Hercules, CA, USA) was used for imaging.

### 2.8. Antioxidant Enzyme Activity

SOD activity was measured using the Ukeda et al. method [33]. CAT was measured using the method of Aebi [34]. GPx was measured using the Paglia and Valentine method [35]. Malondialdehyde (MDA) level was measured using the 2-thiobarbituric acid reactive substances assay [36].

### 2.9. Statistical Analysis

Statistical analyses were performed using SPSS (version 23, SPSS, Inc., Chicago, IL, USA) and all data are expressed as mean ± standard error (SE). The significance of the mean differences was analyzed using one-way analysis of variance (ANOVA), followed by Duncan’s multiple range test for comparisons between the groups at *p* < 0.05.

## 3. Results

### 3.1. Effect of CLW on Grip Strength

**Figure 1:**
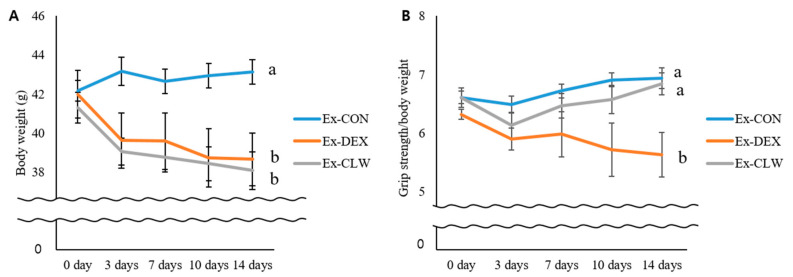
Effect of CLW on the body weight and grip strength in mice with DEX-induced muscle atrophy. Ex-CON, DW administration by gavage and saline IP injection group; Ex-DEX, DW administration by gavage and DEX (1 mg/body weight/day) IP injection group; Ex-CLW, CLW administration by gavage (1 g/body weight/day) and DEX IP injection group. (**A**) Body of the DEX-induced muscle after CLW supplementation in each mouse and (**B**) grip strength/body weight. Data are expressed as mean ± SE for eight mice in each group. Values with different superscripts indicate a significant difference among groups based on Duncan’s multiple range test (*p* < 0.05). CLW may help prevent the decrease in grip strength due to muscle atrophy.

All the mice with DEX-induced muscle atrophy showed weight loss (Figure 1A). The decrease observed in the Ex-CLW group was similar to that in the Ex-DEX group. No significant difference was found between the two groups. As the body weight was reduced, the mice’s grip strength was measured in order to confirm any changes in muscle strength (Figure 1B). Grip strength was not significantly different in all groups before the injection of DEX. Seven days after the DEX injections, the two groups treated with DEX had significantly reduced grip strengths compared to the control group. Between the two groups, the Ex-CLW group had a higher grip strength value than the Ex-DEX group. By day 14, the grip strength in the Ex-DEX group had been found to have consistently decreased since day 7. However, in Ex-CLW group, the grip strength level was similar to that obtained before the injection of DEX. These results indicate that the administration of CLW may help suppress the decrease in grip strength due to muscle atrophy.

### 3.2. Effect of CLW on Muscle Weight

**Figure 2:**
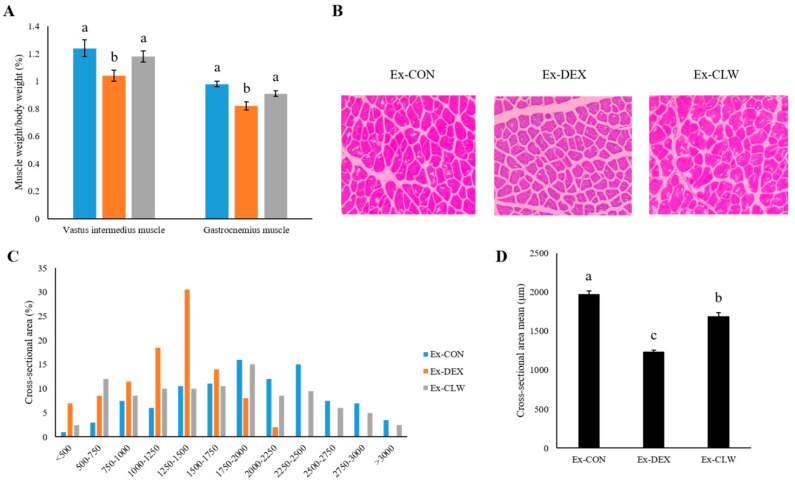
Effect of CLW on DEX-induced muscle atrophy. Ex-CON, DW-supplementation and saline IP injection group; Ex-DEX, DW-supplementation and DEX (1 mg/body weight/day) IP injection group; Ex-CLW, CLW supplementation (1 g/body weight/day) and DEX IP injection group. (**A**) Relative weight of the DEX-induced muscle after CLW supplementation in each mouse. (**B**) Representative hematoxylin and eosin-stained section of the gastrocnemius muscle. Images were analyzed by microscopy and the cross-sectional area (CSA) was measured (×200). (**C**) Distribution of the muscle fiber CSA. (**D**) Cross-sectional area mean. Data are expressed as mean ± SE for eight mice in each group. Values with different superscripts indicate a significant difference among groups based on Duncan’s multiple range test (*p* < 0.05).

The weights of the vastus intermedius and gastrocnemius muscles were measured in each group (Figure 2A). When DEX was injected to the mouse, the amount of muscle was drastically reduced. Conversely, the administration of CLW inhibited the decrease in muscle mass. The muscle weights of the Ex-CLW group were not significantly different from those of the Ex-CON group. The gastrocnemius muscle CSA was measured by H&E staining. As shown in Figure 2B–D the Ex-CLW group had a higher CSA than the Ex-DEX group.

### 3.3. Effect of CLW on Muscle Atrophy-Related mRNA and Protein Expression

**Figure 3:**
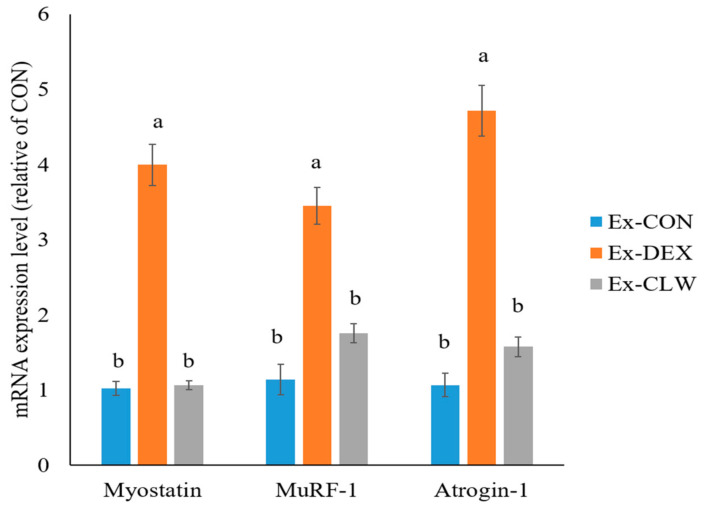
Effect of CLW on the mRNA expression level of the muscle atrophy gene. RT-PCR analysis of the muscle atrophy genes, myostatin, MuRF-1, and Atrogin-1 in the gastrocnemius muscle. Ex-CON, DW supplementation, and saline IP injection group; Ex-DEX, DW-supplementation and DEX (1 mg/body weight/day) IP injection group; Ex-CLW, CLW supplementation (1 g/body weight/day), and DEX IP injection group. Data are expressed as mean ± SE for eight mice in each group. Values with different superscripts indicate a significant difference among groups based on Duncan’s multiple range test (*p* < 0.05).

Muscle atrophy is regulated by myostatin and the MuRF-1 and Atrogin-1 genes. The mRNA expression levels of myostatin, MuRF-1, and Atrogin-1 were significantly increased by the administration of DEX (Figure 3). However, the CLW treatment inhibited these mRNA expressions and their levels were similar to those in the control mice.

**Figure 4:**
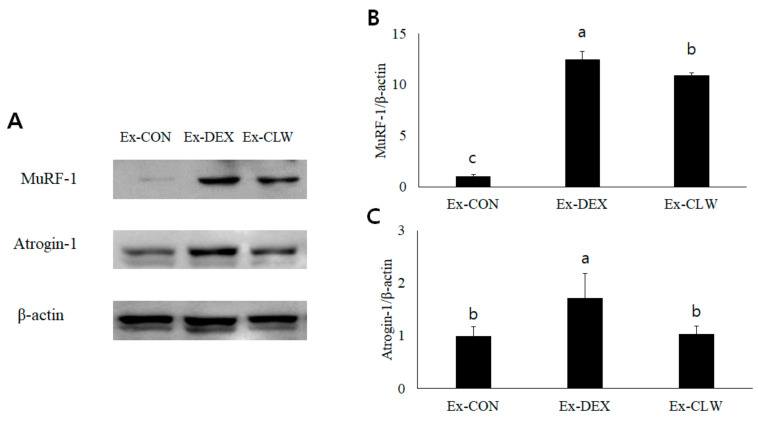
Effect of CLW on MuRF-1 and Atrogin-1 in mice with DEX-induced muscle atrophy. (**A**) MuRF-1 and Atrogin-1 were analyzed by Western blotting. (**B**,**C**) Relative density was calculated as the ratio of MuRF-1 and Atrogin-1 expression to β-actin expression. Ex-CON, DW supplementation, and saline IP injection group; Ex-DEX, DW supplementation, and DEX (1 mg/body weight/day) IP injection group; Ex-CLW, CLW supplementation (1 g/body weight/day), and DEX IP injection group. Data are expressed as mean ± SE for eight mice in each group. Values with different superscripts indicate a significant difference among groups based on Duncan’s multiple range test (*p* < 0.05).

The expression of MuRF-1 and Atrogin-1 was measured by Western blotting. The amount of MuRF-1 and Atrogin-1 protein was found to increase due to DEX (Figure 4). In the Ex-CLW group, the protein levels of MuRF-1 and Atrogin-1 were reduced. This result was similar to that obtained for the mRNA expression levels.

### 3.4. Effect of CLW on Oxidative Stress in Muscle

DEX increases ROS and produces muscle atrophy. To confirm whether DEX increased ROS, the antioxidant enzyme activities and MDA level were measured (Table 1). In the Ex-DEX group, antioxidant enzyme activities decreased while the MDA level increased. However, the Ex-CLW group had similar antioxidant enzyme activities and MDA levels to the Ex-CON group.

## 4. Discussion

Glucocorticoids are a class of steroid hormones used for the treatment of rheumatoid arthritis [37], asthma [38], and various other diseases [39]. Common side effects of glucocorticoids include insomnia, depression, and myopathy. Myopathy is characterized by muscle atrophy, decreased strength, insulin resistance, oxidative stress, and mitochondrial dysfunction [40]. Myopathy caused by these steroids might be due to the use of several drugs such as dexamethasone and betamethasone. In this study, we demonstrated that the administration of CLW prevented DEX-induced muscle atrophy and expression of the ubiquitin proteasome system in mice.

Exercise includes aerobic exercises and resistance exercises. Evidence suggests that resistance exercises can help suppress muscle atrophy [41]. Resistance exercises for animals include electrical stimulation, ladder climbing, and wheel running [42]. Ladder climbing was used for resistance exercises in this study because no stress is caused by electrical stimulation. Additionally, ladder climbing exercises are known to increase muscle strength and mass [43].

The body weight of the mice was reduced by DEX. DEX causes not only muscle atrophy but also side effects such as a loss of appetite [44], fat loss, and weight loss [45]. In previous reports using rodents, the administered curcumin showed no toxicity at 10–2000 mg/kg body weight/day [46]. There was also no change in the body weight when CLW was consumed alone (not shown). Therefore, the body weight of the Ex-DEX group and the Ex-CLW group was reduced. During the experimental period, the grip strength of each group was measured to determine muscle atrophy, thereby allowing the decrease or increase in skeletal muscle mass to be confirmed [13]. Grip strength was more reduced in the Ex-DEX than in the Ex-CON group. The Ex-CLW group had a smaller reduction in grip strength than the Ex-DEX group. CLW supplementation suppressed the muscle atrophy caused by DEX, which helped maintain grip strength. However, it is difficult to identify muscle atrophy with grip strength alone. To confirm the correct muscle loss, the mice were sacrificed to measure their relative muscle weight [47]. This relative muscle weight confirmed that DEX reduced the muscle mass in the Ex-DEX group. When CLW was ingested, the reduction in muscle mass was suppressed. The sacrificed muscle tissue was subjected to H&E staining to confirm the change in fiber size [48]. The muscle fiber size decreased with DEX treatment, and the CSA in the Ex-CLW group was higher than that in the Ex-DEX group. The CLW suppressed muscle atrophy and maintained the CSA of the muscle tissue. This may also have maintained the muscle mass.

DEX activates the ubiquitin proteasome system in muscles by increasing the expression of myostatin [14]. In the ubiquitin proteasome system, MuRF-1 is an E3 ubiquitin ligase that breaks down myosin heavy chains in myopathy [4]. Atrogin-1 is a protein that constitutes E3 ubiquitin ligase and mediates ubiquitination and the subsequent proteasomal degradation of the target protein [49]. MuRF-1 and Atrogin-1, the overexpressed genes that cause muscle atrophy, are activated by myostatin [50]. Myostatin is one of the myokines responsible for inhibiting muscle cell growth and differentiation. As a result, the expression of myostatin, MuRF-1, and Atrogin-1 was confirmed to be increased by DEX. However, the CLW supplementation group decreased the expression of myostatin, thereby reducing the expression of MuRF-1 and Atrogin-1. DEX increased myostatin expression and activated MuRF-1 and Atrogin-1, causing muscle atrophy. However, when CLW was ingested, the expression of myostatin, MuRF-1, and Atrogin-1 was suppressed, ultimately suppressing muscle atrophy. Quercetin and curcumin, which is abundant in *Curcuma longa* L., can be considered the main substance that suppresses muscle atrophy. However, according to Hemdan et al., quercetin does not inhibit the expression of DEX-induced atrogenes [51]. Curcumin is a component that is abundant in *Curcuma longa* L. and is known to suppress muscle atrophy mice with type 1 diabetes and cause muscle atrophy in non-diabetic mice [46]. Despite the presence of many quercetin and curcumin compounds in CLW [20], the quercetin and curcumin in this study’s CLW may have suppressed the expression of DEX-induced atrogenes.

DEX not only increases the expression of the muscle atrophy factor, but also increases ROS to produce muscle atrophy [12]. SOD converts the generated superoxide into H_2_O_2_ and O_2_ [33]. CAT generates non-toxic H_2_O and O_2_, with H_2_O_2_ produced by SOD [34]. GPx also converts the glutathione and H_2_O_2_ produced by glutathione reductase into glutathione disulfide and H_2_O [52]. Lipid peroxidation is the reaction of unremoved ROS with polyunsaturated fatty acids. MDA is produced by lipid peroxidation [53]. By measuring the MDA level and antioxidant enzyme activities, an increase or decrease in the ROS level can be confirmed. The ROS increased in DEX, decreased the antioxidant enzyme activities, and increased the MDA levels in the studied mice. However, the intake of CLW increased antioxidant enzyme activities and decreased the MDA level. This result was similar to that in a previous study where CLW increased antioxidant enzyme activities [54].

## 5. Conclusions

**Figure 5:**
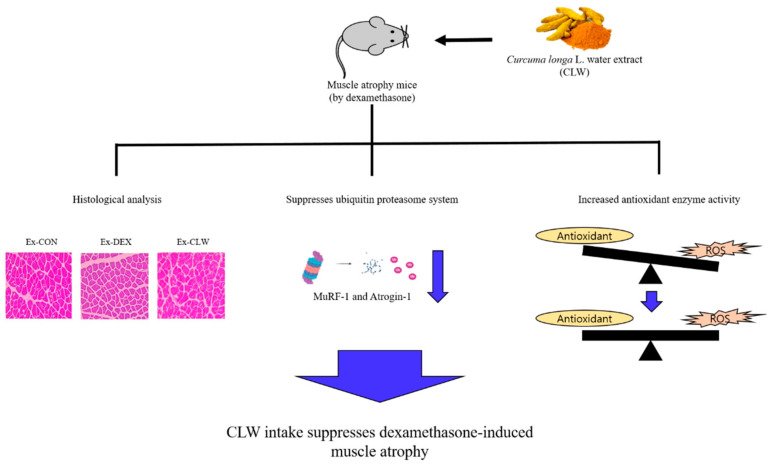
Graphical illustration of how *Curcuma longa* L. water extract improves dexamethasone-induced sarcopenia by modulating the muscle-related gene and oxidative stress in mice.

In conclusion, DEX activates the ubiquitin proteasome system and ROS, causing muscle atrophy. However, CLW ingestion suppresses the ubiquitin proteasome system activated by DEX. Additionally, CLW reduces the levels of ROS increased by DEX. Thus, CLW can suppress DEX-induced muscle atrophy (Figure 5). Since we have confirmed the role of the ubiquitin proteasome system in the various mechanisms that cause muscle atrophy, future studies should investigate muscle atrophy related to autophagy and muscle atrophy caused by aging. The intake of CLW inhibits muscle atrophy, which is expected to prevent a variety of diseases and help an organism to enjoy a healthy life.

## Figures and Tables

**Table 1:** Effect of CLW on muscular antioxidant enzyme activities and MDA level.

Group	Superoxide Dismutase (U/mg Protein)	Catalase (U/mg Protein)	Glutathione Peroxidase (U/mg Protein)	Malondialdehyde (μg/mg Protein)
Ex-CON	27.76 ± 0.94	1.41 ± 0.08	0.30 ± 0.01	0.73 ± 0.11
Ex-DEX	23.33 ± 0.35 *	1.14 ± 0.16 *	0.27 ± 0.01 *	1.02 ± 0.06 *
Ex-CLW	26.85 ± 1.02	1.56 ± 0.22	0.29 ± 0.01	0.65 ± 0.05

Each value represents the mean ± SE of each group. Ex-CON, DW-supplementation, and saline IP injection group; Ex-DEX, DW-supplementation, and DEX (1 mg/body weight/day) IP injection group; Ex-CLW, CLW supplementation (1 g/body weight/day), and DEX IP injection group. The * indicate statistically different values relative to the values of the Ex-CON group as assessed by Duncan’s multiple range test (*p* < 0.05).

## Data Availability

The data presented in this study are available on request from the corresponding author.
